# Burden and Determinants of Anemia Among Rural Adolescent Girls in Andhra Pradesh, India: A Mixed-Methods Study on Nutritional Status, KAP and Stakeholder Insights

**DOI:** 10.3390/ijerph23040424

**Published:** 2026-03-28

**Authors:** Yeswanth Vidyapogu, RamaRao Golime, Venkata Ajay Narendra Talabattula, Vinod Nadella

**Affiliations:** 1 Department of Epidemiology and Public Health, School of Life Sciences, Central University of Tamil Nadu, Thiruvarur 610005, India; 2 Department of Neurology, Nemours Children’s Health, Wilmington, DE 19803, USA; 3 Department of Host Microbe Interactions, St. Jude Children’s Research Hospital, Memphis, TN 38103, USA

**Keywords:** anemia, adolescent health, knowledge, attitude, practices, nutrition, mixed methods, rural health

## Abstract

**Highlights:**

**Public health relevance—How does this work relate to a public health issue?**

- Addresses the high prevalence of anemia and malnutrition among rural adolescent girls in schools from three rural districts of Andhra Pradesh, India, which are a critical and persistent public health concern.
- Utilizes the KAP (knowledge–attitude–practice) framework to uncover gaps in health education, dietary practices, and stakeholder engagement.

**Public health significance—Why is this work of significance to public health?**

- Provides comprehensive insights into multifactorial determinants of anemia and nutrition, combining quantitative and qualitative perspectives.
- Highlights the perspectives and roles of key stakeholders, informing culturally sensitive and context-specific interventions.

**Public health implications—What are the key implications or messages for practitioners, policy makers and/or researchers in public health?**

- Urges the integration of targeted educational programs and participatory approaches to improve adolescent girls’ nutritional outcomes.
- Recommends policy and programmatic shifts to strengthen community involvement and address barriers identified through stakeholder views.

**Abstract:**

**Purpose**: Anemia remains a major public health concern among vulnerable rural adolescent girls in many countries, including India. This study aimed to assess the prevalence of anemia, nutritional status, and anemia-related knowledge, attitudes, and practices (KAP) among school-going rural adolescent girls, along with predictors of KAP score, complemented by stakeholder perspectives. **Methods**: A mixed-methods cross-sectional study was conducted among 553 school-going adolescent girls aged 14–19, selected through a multi-stage stratified random sampling technique from three rural districts of Andhra Pradesh, India. Quantitative data were collected using a structured questionnaire assessing KAP, anthropometric measurements to collect Body Mass Index (BMI) and middle upper arm circumference (MUAC), dietary assessments using a dietary diversity score, and hemoglobin estimation using standardized procedures. Qualitative insights were obtained through focus group discussions (FGDs) with teachers, parents, frontline health workers, and community leaders and analyzed thematically. Logistic regression analysis was performed to identify predictors of KAP. **Results**: The prevalence of anemia among the participants was 55.3%, and 30.7% were underweight. Although over half of the girls demonstrated adequate knowledge of anemia, only 39.6% reported good anemia-preventive practices, indicating a significant gap between knowledge and practice. Dietary scores indicated micronutrient-deficient diet consumption by participants (36.2%), which might be contributing to anemia. Multivariable analysis revealed that maternal education, hemoglobin status, diet patterns, and type of school attended were significantly associated with KAP scores. Qualitative findings highlighted challenges related to health-seeking behavior, cultural misconceptions, gaps in awareness and implementation of existing adolescent health programs. Conclusions: Anemia remains highly prevalent among rural school-going adolescent girls in Andhra Pradesh, with suboptimal anemia-preventive practices despite moderate levels of knowledge. Strengthening school-based nutritional education, improving dietary diversity, and enhancing the reach and effectiveness of adolescent health programs through community engagement may help combat anemia.

## 1. Introduction

Anemia remains a serious public health concern among adolescent girls, adversely affecting their physical growth, energy, learning abilities and school performance, and their future reproductive health [1,2,3]. Adolescence, defined by the World Health Organization (WHO) as between 10 and 19 years, represents a crucial period of human development requiring greater nutritional support for rapid physical and emotional development. In low- and middle-income countries (LMICs), including India, adolescent girls are particularly vulnerable because of unmet increased iron and micronutrient needs during rapid biological and psychosocial development, compounded by menstrual blood loss, infections, food insecurity, and constrained access to nutritious diets and health services [4,5,6]. Global and regional syntheses show that anemia among adolescent girls remains high in South Asia and Sub-Saharan Africa, with slower-than-desired progress in rural areas as rural residence often magnifies these risks through poverty, limited health infrastructure, and gendered intra-household food allocation [7]. India is home to the world’s largest adolescent population, and recent data from the National Family Health Survey-5 (NFHS-5) indicate high and in some regions rising anemia prevalence among adolescent girls, with consistently higher rates in rural than urban areas and substantial interstate variations, underscoring the urgent need for context-specific approaches tailored to rural constraints [8,9,10]. Over the past decade, India has launched several national and regional initiatives pertaining to adolescent nutrition and health, including Anemia Mukth Bharat (AMB), Weekly Iron and Folic Acid Supplementation (WIFS), and Rashtriya Kishore Swastya Karyakram (RKSK) and PM-POSHAN (midday meal). These programs aim to reduce anemia through iron–folic acid supplementation, deworming and behavior change communication, with government schools serving as key delivery platforms, although implementation status and challenges vary across regions [11,12].

Despite these ongoing efforts, anemia continues to persist, especially in rural areas due to gaps in the program coverage and adherence, low dietary diversity, social norms affecting girls’ food allocation, and inconsistent engagement of schools, families, and frontline workers [13,14,15,16,17,18]. Anemia among adolescent girls is not merely a nutritional issue as it also reflects deeper social determinants, including cultural misconceptions, poverty, gender bias and limited access to health services. Several studies have reported that adolescents from lower-income families or with low parental education levels are significantly more likely to be anemic [3]. Similarly, Ghimire et al. (2024) found a significant association between anemia and incomplete IFA supplementation, poor dietary diversity, and socio-economic disadvantage in a region of Nepal [2]. In India, some studies have shown lower anemia rates among girls attending private schools compared to those in government schools, often due to better parental awareness and improved dietary access [6]. Regional research across various states in India has documented wide variability in anemia prevalence; for instance, rates as high as 65.7% have been reported in rural Tamil Nadu [6]. These differences are commonly attributed to variations in dietary habits, socio-economic conditions, access to healthcare, and culturally embedded practices [19,20,21].

Statistics alone may fail to fully capture the real challenges faced by these adolescent girls. While numerous studies in India and other LMICs document the burden of anemia or evaluate the isolated determinants (e.g., hemoglobin levels, BMI, or knowledge score), fewer adopt a mixed-methods approach that simultaneously quantifies anemia prevalence and nutritional status, assesses anemia-related knowledge, attitudes, and practices (KAP) among adolescent girls, identifies predictors of KAP that may inform behavior-change strategies and integrates stakeholders’ perspectives (teachers, parents, health workers, and community leaders) to contextualize barriers to prevention and program uptake in rural India [21,22,23,24]. This integrated approach is essential because improving knowledge does not automatically translate to healthier practices without supportive school, household, and community environments. To address this gap, we conducted a mixed-methods study among school-going rural adolescent girls in three districts of Andhra Pradesh to estimate the prevalence of anemia and described nutritional status, assess anemia-related KAP and examine predictors of KAP scores and synthesize stakeholder insights that may help explain the observed knowledge–practices gap and guide context-appropriate, school- and community-based interventions.

## 2. Methods

### 2.1. Study Population and Design

A mixed-methods cross-sectional study was employed to assess the KAP related to anemia, along with the prevalence of anemia and nutritional status among adolescent girls. A multistage stratified random sampling technique was used to recruit participants from selected schools across three rural districts of Andhra Pradesh, India. Adolescent girls aged 14–19 years enrolled in grades 10, 11, and 12 were invited to participate. The inclusion criteria were: (a) adolescent girls aged 14–19 years; (b) willingness to participate with the ability to provide assent or consent. Girls with chronic illnesses or known hemoglobin disorders were excluded from the study. According to the 2011 census, the population of adolescent girls aged 14–19 years in rural Andhra Pradesh was 2,534,076. The sample size was calculated assuming a 60% anemia prevalence, 95% confidence level, 5% precision, and a design effect of 1.5 to account for clustering, resulting in a final sample of 553 participants.

$$n_{0}=\frac{Z_{1-\alpha /2}^{2}\cdot p(1-p)}{d^{2}}$$

Design effect (DEFF): *n* = *n*_0_ × DEFF

where:

*n* = required sample size;

*Z*_1−*α*/2_ = the *Z*-score corresponding to the desired confidence level (1.96 for 95% confidence);

*p* = anticipated prevalence;

*d* = absolute precision (margin of error).

In the first stage, all schools in the study area were stratified into government and private institutions. From each stratum, schools with eligible participants were randomly selected using a lottery method based on lists obtained from the district educational authorities. In the second stage, within each school selected, adolescent girls from classes 10–12 were chosen in proportion to the school’s enrollment size. School attendance registers served as the sampling frames, and the eligible participants were randomly selected accordingly. A design effect of 1.5 was applied to account for clustering of participants within schools under multistage sampling and the absence of prior intra-cluster correlation estimates.

### 2.2. Data Collection Tools and Procedures

All data collection tools were pre-tested with 20 students from a non-sampled school to ensure questionnaire clarity, validity and reliability. The internal consistency of the KAP items was assessed using Cronbach’s alpha (0.70–0.79), indicating acceptable reliability. Written informed consent or assent was obtained from all participants prior to data collection.

#### 2.2.1. Quantitative Data

Socio-demographic information and KAP data were collected using structured interviewer-administered questionnaires. The anemia-related KAP survey was adapted from validated instruments previously used in national and regional studies [24]. Dietary frequency and diversity were assed using a Food Frequency Questionnaire (FFQ) based on Food and Agriculture Organization (FAO) guidelines [25]. Data collection was carried out over a three-month period (April–June 2025) by trained field investigators. Questionnaires were administered in classroom settings, with investigators available to clarify queries as needed.

The quantitative data collection tools consisted of five components:

1. Socio-demographic profile: Included age, class, school type, family income, mother’s education and occupation, and dietary preference.
2. Knowledge, attitudes, and practices (KAP) on anemia: Assessed knowledge (causes, symptoms, and prevention), attitudes (perceptions and beliefs about anemia and iron supplementation), and practices (dietary habits and IFA tablet use).
3. Food Frequency Questionnaire (FFQ): Evaluated dietary patterns and frequency of consumption of iron-rich foods and anemia-related foods.
4. Anthropometric measurements: Height, weight and mid upper arm circumference (MUAC) were measured by using a portable stadiometer, a digital weighing scale and United Nations Children’s Fund (UNICEF) color-coded MUAC tape. Body Mass Index (BMI) was calculated and classified according to WHO guidelines: normal (18.5–24.9), underweight (<18.5), and overweight (>25). WHO MUAC cutoffs were used to determine nutritional risk and malnutrition.
5. Hemoglobin estimation: Hemoglobin levels were measured using a standardized hemoglobinometer (TrueHb) following aseptic finger-prick procedures. Anemia was classified using WHO cutoff values for adolescent girls (<12 g/dL).

#### 2.2.2. Scoring and Categorization

KAP scores: Participants’ responses were scored and categorized as good, moderate, or poor based on percentile cutoffs for the proportion of correct or positive responses within each KAP domain.

Dietary scores: Dietary intake scores were calculated from the FFQ by summing the reported frequencies of consumption of food items. Total scores were classified as low, moderate, or high dietary intake.

Anemia classification: Hemoglobin levels were categorized according to WHO criteria for adolescent girls as severe anemia (<8.0 g/dL), moderate anemia (8.0–10.9 g/dL), and mild anemia (11.0–11.9 g/dL).

#### 2.2.3. Qualitative Data

FGDs were conducted with key stakeholders including schoolteachers, health workers (Accredited Social Health Activist; ASHA), auxiliary nurse midwives (ANMs), parents or caregivers of adolescent girls, and community leaders in each district. A semi-structured FGD guide was adopted based on a review of the literature [5,16,17,21] and study objectives. The guide was pre-tested with pilot discussions with stakeholders and refined to ensure clarity and relevance. Participants were selected through purposive sampling based on their direct involvement in adolescent health, nutrition, or caregiving. Inclusion criteria required stakeholders to have at least 1–2 years of experience in their respective roles and willingness to provide informed consent. Individuals not directly involved in adolescent health or nutrition, or those unwilling to participate, were excluded. The FGD guide explored key domains such as awareness and perceptions of anemia, barriers to prevention, challenges in implementing anemia prevention programs, adherence to iron–folic acid (IFA) supplementation, the role of schools and families, community beliefs, and opportunities to strengthen adolescent nutrition initiatives. FGDs were conducted by trained moderators with assistance from a note-taker. Each session lasted 45–60 min, was audio-recorded with prior permission, and later transcribed verbatim for analysis. A total of 36 FGDs were conducted among four categories of stakeholders—teachers, parents, health workers, and community leaders. For each stakeholder group, three FDGs were conducted per district. Separate FGDs were held for each category to minimize power dynamics, with 6–8 participants in each group to encourage open discussion. Data from all FGDs were integrated during interpretation and triangulated with quantitative findings to enhance the depth and validity of the analysis.

### 2.3. Statistical Analysis

Quantitative data were analyzed using SPSS software (version 25). Descriptive statistics, including frequencies, means, and standard deviations, were applied to summarize participant characteristics. Associations between categorical variables were assessed using the Chi-square test. Multivariable logistic regression was conducted to identify predictors of good KAP, anemia status, and school type. The results are reported as adjusted odds ratios (AORs) with 95% confidence intervals, and statistical significance was set at *p* ≤ 0.005. Qualitative data from the FGDs were analyzed thematically using Braun and Claske’s six-step thematic analysis approach. Audio recordings were transcribed verbatim and translated from the local language (Telugu) into English for analysis. Coding was performed systematically, and codes were organized into categories and higher-order themes capturing systemic, socio-cultural, dietary, and health-system-related factors influencing anemia. Themes were compared across stakeholder groups to identify convergent and divergent perspectives, determinants of anemia, barriers to prevention, and opportunities for strengthening adolescent nutrition programs.

## 3. Results

### 3.1. Socio-Demographic Characteristics

A total of 553 adolescent girls participated in the study, with a mean age of 16.2 years (SD ± 1.3). The majority of the participants belonged to low-income households, and a substantial proportion of mothers had no formal education (42%) or had completed only primary schooling (38.1%) (Table 1).

### 3.2. Anemia Prevalence

**Figure 1:**
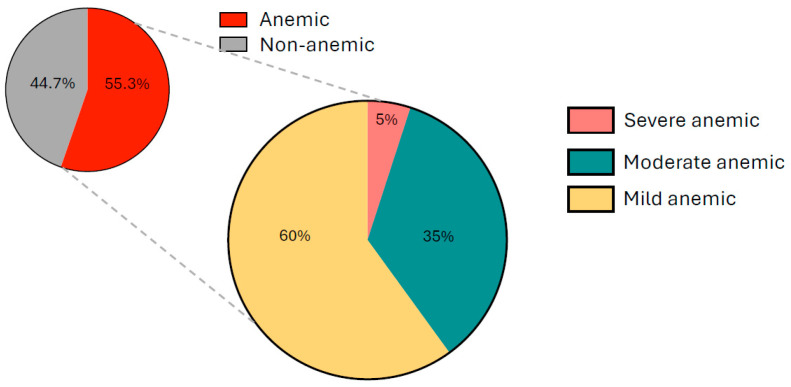
Anemia prevalence among rural adolescent girls (*n* = 553) in 3 districts of Andhra Pradesh, India.

Overall, 55.3% of participants were anemic, while 44.7% were non-anemic (Figure 1). Among those with anemia, 33.3% had mild anemia (11.0–11.9 g/dL), 19.2% had moderate anemia (8–10.9 g/dL), and 2.8% had severe anemia (<8.0 g/dL), according to the WHO classification (Figure 1). Notably, girls attending government schools had a significantly lower prevalence of anemia compared to those in private schools (*p* < 0.001).

### 3.3. Knowledge, Attitude, and Practices Regarding Anemia

**Figure 2:**
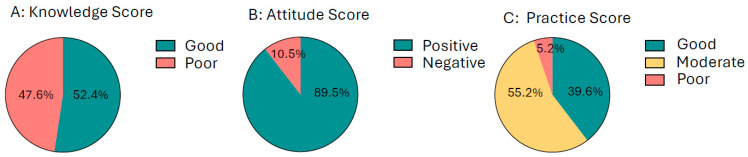
(**A**) Knowledge, (**B**) attitude and (**C**) practice scores among rural adolescent girls (*n* = 553) in 3 districts of Andhra Pradesh, India.

Knowledge: Awareness of anemia was limited, with only 51% of participants reporting that they had heard of the condition. Overall, 52.4% demonstrated good knowledge, while 47.6% had poor knowledge about anemia (Figure 2A). Although 87.2% of the participants were aware of preventive measures and 91.7% could identify common iron-rich foods such as green leafy vegetables, meat, and legumes, deeper understanding was lacking. Only 34.5% were aware of iron absorption enhancers such as vitamin C, and 64.7% recognized inhibitors like tea or calcium. Awareness of IFA supplementation was evenly distributed across participants, indicating mixed levels of familiarity with the program.

Attitudes: A positive attitude towards iron-rich foods was observed among 60.4% of girls; however, only 31.1% reported regular consumption of vitamin C-rich foods. Overall, 89.5% of participants demonstrated positive attitudes towards anemia prevention, reflecting generally favorable perception and willingness to adopt anemia-preventive measures (Figure 2B).

Practices: Hygiene practices were generally good, with 66% reporting regular handwashing and 87.3% wearing slippers. However, only 52.3% of participants had consumed WIFA (Weekly Iron–Folic Acid supplementation) tablets, and 11.6% were unaware of them. Deworming practices were inconsistent; 40.7% of the participants had been dewormed only once, 33.6% twice, and 25.7% had never undergone deworming. Most girls (55.2%) demonstrated moderate preventive practices, with only 39.6% displaying good practices (Figure 2C). Routine hemoglobin monitoring was limited, with just 30.2% undergoing regular testing every 3–6 months, whereas 39.6% had never been tested or were only tested when symptomatic.

### 3.4. Dietary Patterns

**Figure 3:**
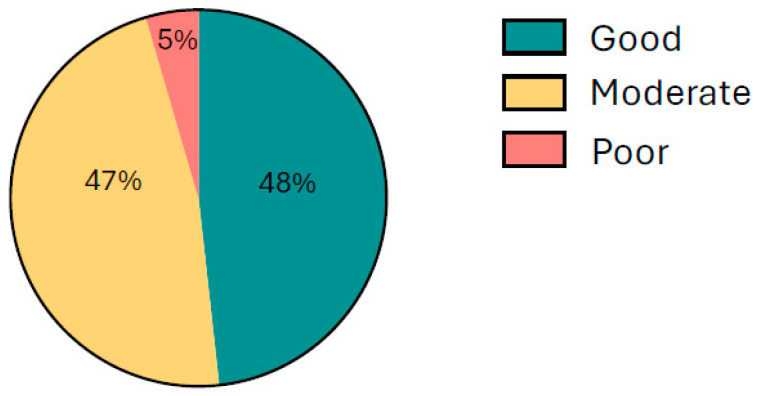
Dietary pattern scores among rural adolescent girls (*n* = 553) in 3 districts of Andhra Pradesh, India.

Dietary assessments indicated that although most participants consumed a calorie-adequate diet, it was largely micronutrient-deficient, characterized by frequent intake of staple and plant-based foods and low consumption of animal-source foods and fruits. Overall, 36.2% of participants had a poor dietary score, reflecting insufficient intake of iron- and vitamin C-rich foods (Figure 3).

### 3.5. Anthropometric and Hemoglobin Parameters

**Figure 4:**
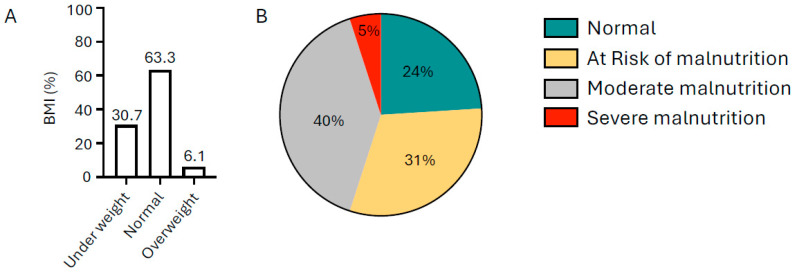
Nutritional status of rural adolescent girls (*n* = 553) in 3 districts of Andhra Pradesh, India, based on BMI (**A**) and MUAC (**B**) scores.

The mean BMI of participants was 20.24 kg/m^2^, which falls within the normal range (18.5–24.9 kg/m^2^), although cases of severe undernutrition were noted, with the lowest recorded BMI being 10.50 kg/m^2^. The mean hemoglobin concentration was 10.52 g/dL, below the WHO cutoff for adolescent girls (<12.0 g/dL) (Table 2). BMI assessment showed that 30.7% of participants were underweight, while 63.3% had a normal BMI. Additionally, 40.5% were classified as at risk of malnutrition based on MUAC measurements (Figure 4A,B).

### 3.6. Associations Among KAP, Socio-Demographics, and Health Measures

**Figure 5:**
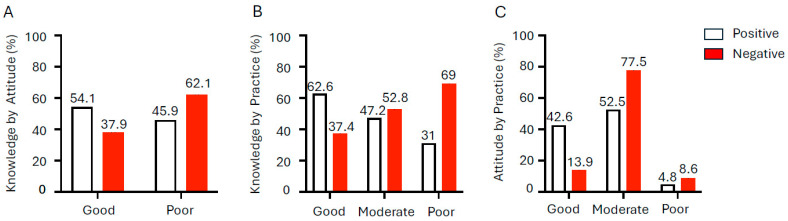
Association between knowledge, attitude and practice among rural adolescent girls (*n* = 553) in three districts of Andhra Pradesh, India. Participants with a positive attitude had a higher proportion of good knowledge compared to those with a negative attitude (**A**). Similarly, practice scores showed significant association with both knowledge (**B**) and attitude (**C**), underscoring the interconnected nature of knowledge, attitude, and practice in shaping adolescent health behavior.

KAP Interrelationships: A significant association was observed between attitude and knowledge scores among the participants (χ^2^ = 5.47, df = 1, *p* = 0.019). Overall, 62% of girls demonstrated good KAP, while 38% fell into the moderate category (Table 2). Participation with a positive attitude had a higher proportion of good knowledge (54%) compared to those with a negative attitude (37.9%) (Figure 5A). Practice scores also showed a significant association with both knowledge (χ^2^ = 17.66, df = 2, *p* < 0.001) and attitude scores (χ^2^ = 18.22, df = 2, *p* < 0.001 (Figure 5B,C). Among girls with good practice scores, 96.3% exhibited a positive attitude, whereas only 3.7% showed a negative attitude. A significant linear trend (χ^2^ = 16.67, df = 1, *p* < 0.001) further confirmed that improved practices were positively correlated with more favorable attitudes. These findings underscore the interconnected nature of knowledge, attitude, and practice in shaping adolescent health behavior.

### 3.7. Socio-Demographic Factors

KAP scores showed significant associations with several socio-demographic variables (Table 3). School type was strongly associated with KAP level (χ^2^ = 49.02, *p* < 0.001), with 76.3% of girls from government schools demonstrating good KAP compared to 47.4% from private schools. Maternal education was also significantly associated with KAP score (χ^2^ = 46.82, *p* < 0.001); girls whose mothers had at least primary or high school education exhibited higher rates of good KAP (75.3%), consistent with evidence showing the intergenerational influence of maternal health literacy [18,19]. Mother’s occupation similarly showed a significant association (χ^2^ = 10.03, *p* = 0.018), with better KAP observed among daughters of skilled workers/professionals. Dietary preference was another significant factor (χ^2^ = 16.06, *p* < 0.001), with vegetarians showing a higher rate of good KAP (75.2%) than non-vegetarians.

### 3.8. Biological and Nutritional Measures

KAP scores were significantly associated with both BMI (χ^2^ = 18.76, *p* < 0.001) and hemoglobin status (χ^2^ = 35.48, *p* < 0.001). Non-anemic girls and those with abnormal BMI (either underweight or overweight/obese) demonstrated comparatively better KAP scores. Specifically, 75.7% of non-anemic girls had good KAP scores, compared to 51% of their anemic peers. Although diet quality showed a positive trend towards higher KAP scores, the association did not reach statistical significance (*p* = 0.078). No significant association was observed between KAP and MUAC (Table 4).

### 3.9. Multivariable Logistic Regression Analysis

A multivariable binary logistic regression model identified several predictors of good KAP (Table 5). Government school attendance was associated with nearly threefold higher odds of good KAP (odds ratio (OR) = 2.89; *p* < 0.001). Maternal education also showed a strong positive association (OR = 2.29; *p* < 0.001). Dietary preference was influential, with vegetarian participants exhibiting higher odds of good KAP (OR = 2.75; *p* < 0.001). Non-anemic girls had significantly higher odds of good KAP (OR = 2.76; *p* < 0.001). In contrast, higher BMI was associated with reduced odds of good KAP (OR = 0.57; *p* = 0.005). Age, grade, mother’s occupation, income, and MUAC were insignificant predictors.

After controlling for factors such as IFA intake, deworming practices, hemoglobin status, and dietary quality, students with good KAP were more likely to attend private schools (OR = 2.26; *p* = 0.004). This finding suggests that the unadjusted advantage observed among students attending government schools may be largely attributable to greater exposure to public health programs and government-supported interventions, rather than inherent differences between school types.

### 3.10. Focus Group Discussions: Stakeholders’ Perspectives

Thematic analysis of FGDs with teachers, healthcare workers, parents, and community leaders identified seven major themes related to anemia awareness and prevention among adolescent girls.

Theme 1: Knowledge and Perceptions of Anemia:

Awareness levels varied considerably across stakeholder groups. Teachers and health workers were generally able to recognize common signs of anemia, such as fatigue, poor concentration, and reduced participation in school activities among girls, while many families often underestimated the seriousness of these symptoms. Parents often attributed tiredness or weakness to normal growth processes or menstruation rather than anemia. Community leaders acknowledged anemia as an issue but frequently expressed generalized or culturally influenced views such as associating adolescent weakness with dietary habits or modern lifestyle factors, including mobile phone use. Although most teachers and health workers demonstrated basic awareness of anemia symptoms, misconceptions about dietary sources and prevention practices remained common among parents.

“*Many parents think weakness is normal in girls at this age and don’t perceive anemia as a disease*”.

(health worker)

“*Yes, we see girls looking weak and inattentive, especially after lunch or during sports periods*”.

(teacher)

Theme 2: Dietary Practices and Challenges with the School Midday Meal:

Across all stakeholder groups, poor dietary diversity emerged as a major concern. Teachers noted that many students had limited awareness of iron-rich foods and raised concerns about the monotony, low palatability, and lack of variety in school midday meals. Healthcare workers attributed the poor nutritional status of adolescent girls to an overreliance on rice, inadequate vegetable intake, and increasing consumption of processed and junk foods, emphasizing the need for parental education. Parents reported difficulty in providing iron-rich foods due to financial constraints. Community leaders expressed concerns about food insecurity and the declining availability of traditional nutrient-dense foods such as pulses and green leafy greens. These qualitative insights align with qualitative findings showing low dietary diversity scores, particularly among girls from lower socio-economic households, where financial limitations directly influence dietary choices and access to iron-rich foods.

“*Students knew about unhealthy foods (chips and noodles), but not about iron rich foods like leafy vegetables*,”

noted a teacher

“*We want to give fruits and meat, but they are expensive and not always available in our village*,”

explained one parent

“*Between school fees and food, we afford what we can*,”

added a father

Stakeholders consistently highlighted the monotony and limited dietary diversity in school midday meals, affecting taste, acceptability and nutritional adequacy.

“*The same rice and curry every day make children lose interest in midday meals; iron-rich foods are rarely included*,”

a teacher remarked

Theme 3: Barriers and facilitators of anemia prevention:

Stakeholders identified several barriers that hinder timely diagnosis, treatment, and prevention of anemia among adolescent girls. A major challenge was delayed disclosure of symptoms. Students often reported weakness, fatigue, or dizziness only when symptoms became visibly severe or when school attendance was affected. Access to health services was largely dependent on school-based health programs, with many girls seeking care only during scheduled visits rather than proactively. Financial constraints, long waiting times at public health facilities, and travel difficulties further contributed to reliance on unqualified or informal health providers. Poor compliance with IFA supplementation emerged as a recurrent barrier. Myths regarding side effects, such as nausea and stomach pain, or beliefs that tablets cause “body heat” or weight gain, led many girls to avoid or discard IFA tablets. Limited awareness about the importance of consistent supplementation and inadequate follow-up further compounded these challenges. Stakeholders noted that delayed diagnosis was closely linked to poor health-seeking behavior, irregular screening, and a lack of systemic monitoring.

“*Parents usually bring girls only when symptoms become severe*”.

(health worker)

“*Girls throw away the tablets or complain of nausea or bitter taste*”.

(another health worker)

Theme 4: Role of schools, health programs, and community engagement:

Stakeholders highlighted inconsistencies in the implementation of school-based health programs, particularly irregular IFA distribution and limited follow-up. Although teachers acknowledge the presence of programs such as WIFS, they noted that implementation varied widely across schools. Community leaders similarly expressed concerns about the limited frequency and continuity of community-based efforts, including health camps and Anganwadi services, which reduced their long-term effectiveness. Variability in school cooperation with healthcare personnel was also reported, with some prioritizing academic schedules over health activities.

“*Some schools are supportive, while others are too focused on academics.*”

(Health supervisor)

Theme 5: Cultural norms, myths, and social influences:

Deep-rooted cultural beliefs emerged as a major barrier to effective anemia management. Myths surrounding IFA supplementation, such as fear of infertility, excessive body heat, or adverse side effects discouraged consistent use. Traditional dietary taboos during menstruation, including avoidance of foods like papaya, tamarind, or certain leafy vegetables further limited nutrient intake. Older family members often dismissed anemia-related symptoms as routine, reinforcing neglect of adolescent health needs. Reluctance to openly discuss girls’ health within families also perpetuated low awareness and delayed care seeking.

“*Families hesitate to talk about girls’ health or think it’s not important*”.

(community elder)

“*We spend half the time just convincing them that tablets are safe*”.

(healthcare worker)

These findings highlight the strong influence of socio-cultural beliefs on adolescent health behaviors and the importance of sensitizing households and communities as part of anemia-preventive programs.

Theme 6: Structural challenges and systemic barriers:

Stakeholders identified several structural and systemic challenges that hinder implementation of anemia-preventive efforts. Teachers reported insufficient training, limited time and lack of appropriate educational materials, which constrained their ability to integrate nutrition education into classroom activities. Inadequate and irregular IFA supply and distribution were also frequently cited, contributing to poor program continuity. Low attendance at community outreach activities and the absence of standardized tracking or follow-up mechanisms further weakened program effectiveness. Parents repeatedly emphasized financial constraints as a barrier to accessing nutritious food and healthcare services, while community leaders pointed to the broader influence of poverty and entrenched gender norms on girls’ health-seeking behavior. These structural vulnerabilities collectively limit adolescents’ access to timely diagnosis, counseling, and consistent supplementation.

“*We are not given proper content or sessions to teach about health education*”.

(teacher)

Theme 7: Recommendations and suggestions by stakeholders:

Stakeholders proposed several strategies to strengthen anemia prevention initiatives for adolescent girls. Key recommendations included integrating structured nutritional education into the school curriculum and using cultural events, school functions, and parent–teacher meetings as platforms for awareness building. Improved communication between schools and families were emphasized to ensure consistent messaging about diet, supplementation, and health-seeking practices. Stakeholders also highlighted the importance of fostering collaboration between health departments, NGOs and local community leaders to enhance program reach, ensure reliable IFA distribution, and promote community-wide engagement in anemia-preventive efforts.

## 4. Discussion

This study provides an integrated understanding of anemia-related KAP among rural high-school-going adolescent girls using a mixed-methods study. The findings reveal a high prevalence of anemia, suboptimal dietary diversity, limited awareness regarding iron absorption, and persistent misconception surrounding IFA supplementation. Although more than half of the participants demonstrated good knowledge, attitudes and preventive practices were comparatively weaker, highlighting a significant knowledge–practice gap. Quantitative data further contextualized these findings by illustrating the social, cultural, and structural barriers shaping health behavior.

The prevalence of anemia (55.3%) observed in this study aligns with earlier studies from India, which report anemia rates ranging from 45% to 65% among adolescent girls [8,9,10,15]. At the national level, NFHS-5 documented an anemia prevalence of 59.1% among girls aged 15–19 years, suggesting that the burden in this study area mirrors national patterns. Globally, adolescent anemia prevalence varies widely, with South Asia and African countries reporting some of the highest rates primarily due to poor dietary diversity, menstrual blood loss, and high infection rates [7,26,27,28,29,30]. The findings therefore reaffirm anemia as a regional and global public health challenge disproportionately affecting adolescent girls. Knowledge levels in the present study (52.4%) were comparable to earlier studies that reported moderate awareness of anemia symptoms and risk factors but limited understanding of micronutrient absorption and IFA supplementation [31,32,33,34,35]. The gap between awareness and practice observed here has been echoed in prior research, which notes that knowledge alone rarely translates into behavior change unless reinforced by supportive environments, community engagement, and continuous program visibility [12,36]. The limited awareness of factors affecting iron absorption and low familiarity with IFA benefits are consistent with evidence showing inadequate program reach and health education in rural settings [5,6,11,12,37].

Maternal education emerged as a strong predictor of KAP performances, supporting evidence that parental education and household health literacy strongly influence adolescent nutritional behaviors and health-seeking patterns [12,37]. The association between positive attitudes and better practices further aligns with previous findings showing attitudes as an important mediator between knowledge and behavior [30,34,35]. Interestingly, government school students had better KAP scores than those in private schools (76.3% vs. 47.4%), likely reflecting better integration and reach of government health programs such as WIFS in government schools. Yet, both quantitative and qualitative findings pointed to gaps in program implementation, including inconsistent distribution of IFA tablets, inadequate counseling and low adherence due to perceived side effects.

Dietary patterns in this study were characterized by calorie-sufficient yet micronutrient-poor diets, mirroring national dietary deficiencies documented among adolescents [12,38]. Limited dietary diversity, high reliance on cereal-based meals, and low intake of iron-rich foods were central themes in both survey responses and focus group discussions. Similar to previous local and national reports, misconceptions about IFA tablets, social stigma related to menstruation, and limited family support emerged as key barriers to adopting healthy practices [11,12,16,17]. Stakeholders’ normalization of fatigue and pallor as part of growth or menstruation has been reported previously [5,17,31] and further delays recognition and timely management of anemia.

The qualitative findings enriched the quantitative results by highlighting structural challenges, including training of teachers and healthcare workers, a lack of teaching aids, and economic constraints limiting dietary improvement. These insights are consistent with the regional literature documenting fragmented implementation of school-based health programs, especially in underserved rural areas. Collectively, these findings underscore the need for multi-level interventions that address environmental, social, and structural determinants of anemia in addition to improving individual knowledge.

## 5. Limitations

While the mixed-methods approach strengthened the comprehensiveness of the study, the cross-sectional design limits casual interference, and self-reported dietary and behavioral data may be influenced by recall or social desirability bias. The study sample was limited to high-school-going rural adolescent girls and therefore may not be generalizable to out-of-school adolescents or other rural adolescent girls. Future studies should include longitudinal or pre- and post-intervention designs to access changes in KAP following targeted educational or nutritional interventions and incorporate objective biomarkers to complement self-reported data.

## 6. Summary, Recommendations and Conclusions

This study identified a high prevalence of anemia and undernutrition among rural adolescent girls, largely driven by inadequate dietary diversity and suboptimal preventive practices, despite good knowledge and attitudes regarding anemia. The quantitative findings show that maternal education, school type, and dietary habits emerged as significant determinants of KAP, while dietary assessment revealed inadequate dietary diversity and limited consumption of iron-rich foods. These patterns indicate that knowledge alone is insufficient to drive appropriate dietary and preventive behavior. Complementing these findings, qualitative insights obtained through FGDs with teachers, parents, frontline health workers, and community leaders identified several further systemic barriers including cultural misconceptions about anemia and supplementations, inconsistent implementations of health programs, poor compliance with IFA tablet intake, and limited community awareness that collectively impede the translation of knowledge into sustained healthy behavioral practices.

Together, the quantitative and qualitative findings underscore that addressing anemia among rural adolescent girls requires integrated, context-specific interventions that engage not only adolescents but also households, schools, and the wider community. Interventions must extend beyond information dissemination and incorporate behavior-focused, culturally sensitive approaches that facilitate practical skill-building and strengthen supportive environments for behavior changes. Grounded in the evidence generated from this study, the following recommendations aim to promote sustainable improvements in anemia prevention and adolescent nutrition:

1. Strengthen behavior-focused nutrition education to close the knowledge–practice gap: School-based education should incorporate practical demonstrations, peer-led activities, and locally relevant examples emphasizing iron-rich foods, enhancers of iron absorption, and reduction in inhibitors, given the identified inadequate dietary diversity.
2. Enhance maternal health literacy and parental engagement: Since maternal education strongly influenced adolescent KAP, community-based learning sessions and parent–teacher engagement activities focused on anemia prevention, dietary practices, and healthy behavior may meaningfully improve outcomes.
3. Improve school-level supervision and counseling for IFA supplementations: Irregular IFA intake reported by participants indicates the need for stronger monitoring, supportive counselling, and myth-busting conversations among teachers and health workers to encourage consistent adherence.
4. Address cultural misconceptions and strengthen community awareness: Misconceptions identified through qualitative findings highlight the need for culturally tailored awareness initiatives led by trusted community members to shift norms and improve acceptance of preventive practices.
5. Promote consumption of locally available, affordable iron-rich foods: Given the low intake of iron-rich foods, coordinated efforts involving parents, teachers and health workers such as cooking demonstrations and home-based nutrition initiatives can improve diet quality within the constraints of local affordability and accessibility.
6. Improve program implementation through regular monitoring, teacher training, and intersectoral coordination: Inconsistencies in program delivery suggest the need for strengthened oversight, enhanced teacher training on adolescent nutrition and anemia, and better coordination between schools, health workers, and community organizations.

In conclusion, this study demonstrates that anemia among school-going rural adolescent girls is driven not only by dietary inadequacies but also by systemic, cultural, and implementation-related barriers that inhibit the translation of knowledge into healthy practices. By addressing these multi-level determinants through the evidence-informed strategies outlined above, more effective and sustainable anemia prevention efforts can be realized for this vulnerable population.

## Figures and Tables

**Table 1:** Socio-demographic characteristics of the study participants (*n* = 553).

Variable	Category	Frequency (*n*)	Percentage (%)
Type of School	Government School	279	50.5
	Private School	274	49.5
Age of Students	14–15 years	176	31.8
	16–17 years	189	34.2
	18–19 years	188	34.0
Grade	10th Class	188	34.0
	11th Class	178	32.2
	12th Class	187	33.8
Mother’s Education	No Formal Education	232	42.0
	Primary/High School	271	49.0
	Graduate	50	9.0
Mother’s Occupation	Housewife	344	62.2
	Skilled Worker	98	17.7
	Unskilled Worker	85	15.4
	Professional	26	4.7
Family Annual Income	<₹50,000 (<$550)	46	8.3
	₹50,000–₹100,000 ($550–1100)	257	46.5
	₹100,001–₹200,000 ($1100–2200)	193	34.9
	₹200,001–₹500,000 ($2200–5500)	54	9.8
	₹500,001–₹1,000,000 ($5500–110,000)	3	0.5
Dietary Preference	Vegetarian	157	28.4
	Non-Vegetarian	396	71.6

**Table 2:** Frequency distribution of key study variables (*n* = 553).

Variable	Category	Frequency (*n*)	Percentage (%)
Knowledge Score	Poor	263	47.6%
	Good	290	52.4%
Attitude Score	Negative	58	10.5%
	Positive	495	89.5%
Practice Score	Poor	29	5.2%
	Moderate	305	55.2%
	Good	219	39.6%
BMI (*n* = 551)	Underweight	170	30.7%
	Normal	349	63.3%
	Overweight	34	6.0%
MUAC Score	Normal	131	23.7%
	At risk of malnutrition	224	40.5%
	Moderate malnutrition	169	30.6%
	Severe malnutrition	29	5.2%
Hemoglobin Status	Anemic	306	55.3%
	Non-anemic	247	44.7%
Dietary Score	Poor	25	4.5%
	Moderate	261	47.2%
	Good	267	48.3%
Overall KAP Score	Moderate KAP	210	38.0%
	Good KAP	343	62.0%

**Table 3:** Association between socio-demographic variables and categorized KAP scores on anemia (*n* = 553).

Variable	Category	Moderate *n* (%)	Good *n* (%)	Chi-Square (df)	*p*-Value
Age Group	14–15 years	77 (43.8%)	99 (56.2%)	χ^2^ = 4.15 (2)	0.125
	16–17 years	70 (37.0%)	119 (63.0%)		
	18–19 years	63 (33.5%)	125 (66.5%)		
School Type	Govt. school	66 (23.7%)	213 (76.3%)	χ^2^ = 49.02 (1)	<0.001 ***
	Private school	144 (52.6%)	130 (47.4%)		
Grade Level	10th class	83 (44.1%)	105 (55.9%)	χ^2^ = 4.81 (2)	0.090
	11th class	64 (36.0%)	114 (64.0%)		
	12th class	63 (33.7%)	124 (66.3%)		
Mother’s Education	No formal education	126 (54.3%)	106 (45.7%)	χ^2^ = 46.82 (2)	<0.001 ***
	Primary/High School	67 (24.7%)	204 (75.3%)		
	Graduate	17 (34.0%)	33 (66.0%)		
Mother’s Occupation	Housewife	146 (42.4%)	198 (57.6%)	χ^2^ = 10.03 (3)	0.018 *
	Skilled worker	25 (25.5%)	73 (74.5%)		
	Unskilled worker	31 (36.5%)	54 (63.5%)		
	Professional	8 (30.8%)	18 (69.2%)		
Family Annual income	<₹50,000 (<$550)	15 (32.6%)	31 (67.4%)	χ^2^ = 6.44 (4)	0.168
	₹50k–1 lakh ($550–1100)	90 (35.0%)	167 (65.0%)		
	₹1–2 lakhs ($1100–2200)	79 (40.9%)	114 (59.1%)		
	₹2–5 lakhs ($2200–5500)	26 (48.1%)	28 (51.9%)		
	₹5–10 lakhs ($5500–11,000)	0 (0.0%)	3 (100.0%)		
Dietary Preference	Vegetarian	39 (24.8%)	118 (75.2%)	χ^2^ = 16.06 (1)	<0.001 ***
	Non-vegetarian	171 (43.2%)	225 (56.8%)		

* *p* < 0.05; *** *p* < 0.001

**Table 4:** Association between biological/nutritional measures and KAP category (*n*= 553).

Variable	Category	Moderate *n* (%)	Good *n* (%)	Chi-Square (df)	*p*-Value
BMI	Underweight	43 (25.3%)	127 (74.7%)	χ^2^ = 18.76 (2)	<0.001 ***
	Normal	156 (44.7%)	193 (55.3%)		
	Overweight/obese	11 (32.4%)	23 (67.6%)		
MUAC	Normal	49 (37.4%)	82 (62.6%)	χ^2^ = 0.16 (3)	0.984
	At risk of malnutrition	85 (37.9%)	139 (62.1%)		
	Moderate malnutrition	64 (37.9%)	105 (62.1%)		
	Severe malnutrition	12 (41.4%)	17 (58.6%)		
Hemoglobin Status	Anemic	150 (49.0%)	156 (51.0%)	χ^2^ = 35.48 (1)	<0.001 ***
	Non-anemic	60 (24.3%)	187 (75.7%)		
Diet Score	Poor	12 (48.0%)	13 (52.0%)	χ^2^ = 5.10 (2)	0.078
	Moderate	109 (41.8%)	152 (58.2%)		
	Good	89 (33.3%)	178 (66.7%)		

*** *p* < 0.001.

**Table 5:** Multivariable logistic regression predicting good KAP (*n* = 553).

Predictor	B	SE	Wald	df	*p*-Value	OR (Exp[B])	95% CI for OR
Age of students	0.344	0.631	0.297	1	0.586	1.411	0.409–4.863
Type of school (private)	−1.057	0.217	23.640	1	<0.001	0.347	0.227–0.532
Study grade	−0.001	0.620	0.000	1	0.999	0.999	0.296–3.371
Mother’s education	0.828	0.171	23.438	1	<0.001	2.289	1.637–3.201
Mother’s occupation	−0.048	0.123	0.156	1	0.693	0.953	0.749–1.211
Family annual income	−0.001	0.131	0.000	1	0.994	0.999	0.773–1.291
Dietary preference	−1.011	0.255	15.764	1	<0.001	0.364	0.221–0.599
BMI category	−0.561	0.201	7.824	1	0.005	0.571	0.385–0.845
MUAC category	−0.180	0.123	2.131	1	0.144	0.835	0.656–1.064
Hemoglobin status	1.014	0.214	22.399	1	<0.001	2.757	1.812–4.197
Diet score	0.567	0.190	8.963	1	0.003	1.764	1.216–2.557
Constant	0.497	0.963	0.267	1	0.605	1.645	–

## Data Availability

The original contributions presented in this study are included in the article. Further inquiries can be directed to the corresponding authors.
